# Multi-drug resistant pathogenic bacteria in the gut of young children in Bangladesh

**DOI:** 10.1186/s13099-017-0170-4

**Published:** 2017-04-20

**Authors:** Shirajum Monira, Syeda Antara Shabnam, Sk. Imran Ali, Abdus Sadique, Fatema-Tuz Johura, Kazi Zillur Rahman, Nur Haque Alam, Haruo Watanabe, Munirul Alam

**Affiliations:** 1 International Center for Diarrheal Disease Research, Bangladesh (icddr,b), 68 Shaheed Tajuddin Ahmed Sarani, Mohakhali, Dhaka, 1212 Bangladesh; 2 National Institute of Infectious Diseases, Tokyo, Japan

**Keywords:** Children, Gut, Microbiota, Multidrug resistance, ESBL related genes

## Abstract

**Background:**

The gut of human harbors diverse commensal microbiota performing an array of beneficial role for the hosts. In the present study, the major commensal gut bacteria isolated by culturing methods from 15 children of moderate income families, aged between 10 and 24 months, were studied for their response to different antibiotics, and the molecular basis of drug resistance.

**Results:**

Of 122 bacterial colonies primarily selected from Luria–Bertani agar, bacterial genera confirmed by analytical profile index (API) 20E^®^ system included *Escherichia* as the predominant (52%) organism, followed by *Enterobacter* (16%), *Pseudomonas* (12%), *Klebsiella* (6%), *Pantoea* (6%), *Vibrio* (3%), and *Citrobacter* (3%); while *Aeromonas* and *Raoultella* were identified as the infrequently occurring genera. An estimated 11 and 22% of the *E. coli* isolates carried virulence marker genes *stx*-2 and *eae*, respectively. Antimicrobial susceptibility assay revealed 78% of the gut bacteria to be multidrug resistant (MDR) with highest resistance to erythromycin (96%), followed by ampicillin (63%), tetracycline (59%), azithromycin (53%), sulfamethoxazole-trimethoprim (43%), cefixime (39%), and ceftriaxone (33%). PCR assay results revealed 56% of the gut bacteria to possess gene cassette Class 1 integron; while 8, 17.5 and 6% of the strains carried tetracycline resistance-related genes *tet*A, *tet*B, and *tet*D, respectively. The macrolide (erythromycin and azithromycin) resistance marker genes *mph*A, *ere*B, and *erm*B were found in 28, 3 and 5% of bacterial isolates, respectively; while 26, 12, 17, 32, 7, 4 and 3% of the MDR bacterial isolates carried the extended spectrum β-lactamase (ESBL)-related genes e.g., *bla*_TEM_, *bla*_SHV_, *bla*_CMY-9_, *bla*_CTX-M1_, *bla*_CTX-M2_, *bla*_CMY-2_ and *bla*_OXA_ respectively. Majority of the MDR gut bacteria harbored large plasmids [e.g., 140 MDa (43%), 105 MDa (30%), 90 MDa (14%)] carrying invasion and related antibiotic resistance marker genes.

**Conclusions:**

Our results suggest gut of young Bangladeshi children to be an important reservoir for multi-drug resistant pathogenic bacteria carrying ESBL related genes.

## Background

The gut of human harbors diverse microbial community including 400–500 different species of bacteria performing an array of important metabolic functions for the host namely production of short-chain fatty acids and vitamins (vitamin k and biotin), nutrient absorption, and fighting against invading pathogens [1, 2]. Diarrhea and poor child health are longstanding problems for many developing countries where sanitation is poor and the source of safe drinking water is scarce. In Bangladesh, a child under 5 years of age frequently suffers from moderate to severe diarrhea, on an average 3–4 times a year [3]. The hospitalized patients suffering from severe diarrhea (cholera) undergo rehydration therapy and a 3 day course of effective antibiotic to shorten the hospitalization and to prevent the spread of infectious bacteria into the environment [4, 5]. Although antibiotic therapy prevents people from death due to severe infection and related dehydration, the effect of antibiotics on the gut flora in acute diarrhea remains an important area to explore.

Our culture-independent metagenomic studies provided evidence that acute diarrhea (cholera) results in the expulsion of most of the commensal gut microbiota, and drug therapy allows the drug-resistant pathogenic microbiota to colonize the gut [7]. Our metagenomic studies also revealed that the members of the phyla Proteobacteria including pathogenic genera were predominant in the gut of malnourished children compared to their healthy counterparts [6]. In Bangladesh, infectious diseases due to multidrug resistant (MDR) bacteria are at rise, although the reservoir for such MDR bacteria is not well defined. This study, a follow up, was designed to analyze gut microbiota from 15 children aged between 10 and 24 months, who did not have immediate past history of diarrhea for last 2 months, and were from moderate income families living in moderate hygiene condition in Dhaka city. We confirmed the gut of children under 5 years of age as an important reservoir for MDR bacterial genera belonging to the family Enterobacteriaceae, which included surface water bacteria, including important human pathogens carrying major virulence plasmids and related genetic elements responsible for multidrug resistance.

## Methods

### Study subjects

This study was conducted in Dhaka city during February 2011–July 2012 on gut bacteria of 15 non-diarrheal children from moderate socioeconomic status. The age of the children ranged between 10 and 24 months and their weight for height was >100% according to the National Centre for Health Statistics Median [8]. All children were residing in their home, without any sign of ailments, and none had any history of taking any antibiotics during the past 2 months. Their baseline characteristics are presented in Table 1. For sample collection, health workers visited the house-holds of the children to get mothers’ consents and to demonstrate the sample collection procedure by providing them pots for stool collection. Fresh stool samples collected in the following morning were transferred to the laboratory maintaining cold chain.

**Table 1:** Baseline characteristics of study children

Criteria	Healthy children (n = 15)
Age, month	20.9 ± 6.4
Weight, kg	11.8 ± 1.0
Height, cm	74.9 ± 2.9
Weight/height, %	114.5 ± 5.9
Weight/age, %	101.3 ± 3.1
Male/female	9/6
Socio-economic status	Moderate

Values are expressed as mean ± SD

### Enrichment, plating and identification of bacteria

Stool samples were inoculated for enrichment in 3 ml of Luria Broth (Difco, USA) using sterile cotton swabs and incubated overnight at 37 °C in a shaking water bath. The enriched samples were diluted six folds and streaked onto Luria Agar (Difco, USA) plates; and the growth of the colonies were observed after overnight incubation at 37 °C. Twenty colonies of various morphological types (viz., size, shapes, elevation, opacity) representing bacterial community were randomly selected from each samples and identified by culturing over night in the Analytical Profile Index (API 20E^®^) test kit (bioMerieux Inc., Hazelwood, MO).

### Antibiotic susceptibility testing

Antimicrobial susceptibility assay of the bacterial strains was performed using agar disc diffusion assay method recommended by the Clinical and Laboratory Standards Institute [9]. Commercially available antimicrobial discs (Mast Diagnostics, U.K) of erythromycin (15 µg), gentamicin (10 µg), sulfamethoxazole-trimethoprim (25 µg), tetracycline (30 µg), ciprofloxacin (5 µg), azithromycin (15 µg), ampicillin (10 µg), ceftriaxone (30 µg), cefixime (5 µg), and mecillinam (10 µg) were placed on Mueller–Hinton agar (Bio-Rad) seeded with bacteria cultured in their early log phase, as previously described CLSI [9]. Following incubation, the plates were examined, and the inhibitory zone diameters for individual antimicrobial agents were measured and recorded as susceptible and resistant based on the breakpoints for respective antibiotic susceptibility of CLSI (CLSI 2010). Organisms resistant to 3 or more classes of antibiotics are designated as MDR. Zone diameter, breakpoints (mm): erythromycin (15 μg) S-I-R: >26, 23–25, <2; gentamicin (10 μg): >15, 13–14, <12; sulfamethoxazole trimethoprim (25 μg): >26, 23–25, <22; tetracycline (30 μg): >26, 23–25, <22; ciprofloxacin (5 μg): >21, 15–20, <14; ampicillin (10 µg): >17,14–16, <13; ceftriaxone (30 μg): >16, 13–15, <12; cefixime (5 μg): >19, 16–18, <15; azithromycin (15 μg): >18, 14–17, <13; mecillinam (10 μg): >15, 12–14, <11.

### Extraction of genomic DNA and plasmids from bacteria

Genomic DNA was extracted from 3.0 ml of cells cultured overnight in LB broth using alkaline lysis method followed by phenol–chloroform extraction, as described elsewhere [10]. The eluted DNA was stored at −20 °C.

Plasmid DNA was extracted according to the conventional simplified alkaline lysis method [11] and afterwards separated by horizontal electrophoresis in 0.8% agarose gels in a Tris–borate EDTA buffer at room temperature at 100 V (50 mA) for 3 h. Thirty microliters of plasmid DNA solution were mixed with 3 µl of tracking dye and was loaded into the individual well of the gel. The gel was stained with ethidium bromide (0.1 ng/ml) for 30 min at room temperature. DNA bands were visualized and photographed using Gel Documentation with UV Trans illuminator. The molecular weight of the unknown plasmid DNA was determined on the basis of its mobility through agarose gel and was compared with the mobility of the known molecular weight plasmids present in strains of *E. coli* PDK-9 of sizes 140, 105, 2.7 and 2.1 MDa.

### PCR detection of genes associated with virulence and antibiotic resistance of *E. coli*

*Escherichia coli* strains isolated and confirmed by API 20E^®^ were screened by PCR for the detection of virulence genes namely *stx*1, *stx*-2, *lt*, and *eae* by following methods as described elsewhere [12]. The gut bacteria isolated in present study were screened for macrolide resistance marker genes such as *mph*A, *ere*B and *erm*B [13]; tetracycline resistance genes e.g., *tet*A, *tet*B, and *tet*D [14]; β-lactam resistance gene in enteric bacteria e.g., *bla*_TEM_, *bla*_SHV_, *bla*_OXA_, *bla*_CMY-9_, *bla*_CMY-2_, *bla*_CTX-M1_, and *bla*_CTX-M2_ [14, 15]. The distribution of integrons in multi-drug resistant bacteria was determined by PCR primer targeted against the conserved regions of the integrin-encoded integrase genes, *intl*-1 [16]. SXT is a family of conjugative-transposon-like mobile genetic elements encoding multiple antibiotic resistance genes. Multi-drug resistant bacteria were analyzed for the presence of *SXT*- related genes by PCR targeting the integrase gene (int) of the SXT [17]. The PCR primers used in this study are listed in Table 2.

**Table 2:** PCR primers for screening virulence and antibiotic resistance genes in bacteria isolated from the gut of healthy children in Bangladesh

Primer	Primer sequence (5′ → 3′)	Target	Amplicon size (bp)	References
Stx2f	GGCACTGTCTGAAACTGCTCC	*stx*_2_	225	[12]
Stx2r	TCGCCAGTTATCTGACATTCTG			
eae1	CTGAACGGCGATTACGCGAA	*eae*	917	[12]
eae2	CCAGACGATACGATCCAG			
INT-1U	GTTCGGTCAAGGTTCTG	*intl*1	923	[16]
INT-1D	GCCAACTTTCAGCACATG			
mphA(F)	GTGAGGAGGAGCTTCGCGAG	*mphA*	403	[13]
mphA(R)	TGCCGCAGGACTCGGAGGTC			
ereB(F)	TTGGAGATACCCAGATTGTAG	*ereB*	537	[13]
ereB(R)	GAGCCATAGCTTCAACGC			
ermB(F)	GAAAAAGTACTCAACCAAATA	*ermB*	639	[13]
ermB(R)	AATTTAAGTACCGTTACT			
INT1	GCTGGATAGGTTAAGGGCGG	*Sxt*-*integrase*	592	[17]
INT2	CTCTATGGGCACTGTCCACATTG			
TEM(F)	ATTCTTGAAGACGAAAGGGC	*bla*_TEM_	1150	[14]
TEM(R)	ACGCTCAGTGGAACGAAAAC			
OXA-F	ACACAATACATATCAACTTCGC	*Bla*_OXA_	813	[14]
OXA-R	AGTGTGTTTAGAATGGTGATC			
SHV-F	CACTCAAGGATGTATTGTG	*bla*_SHV_	885	[14]
SHV-R	TTAGCGTTGCCAGTGCTCG			
CMY-9(F)	TCAGCGAGCAGACCCTGTTC	*bla*_CMY-9_	874	[15]
CMY-9(R)	CTGGCCGGGATGGGATAGTT			
CTX-M1(F)	AACCGTCACGCTGTTGTTAG	*bla*_CTX-M1_	766	[15]
CTX-M1(R)	TTGAGGCTGGGTGAAGTAAG			
CTX-M2(F)	GGCGTTGCGCTGATTAACAC	*bla*_CTX-M2_	486	[15]
CTX-M2(R)	TTGCCCTTAAGCCACGTCAC			
TetA (F)	GTAATTCTGAGCACTGTCGC	*tetA*	937	[14]
TetA (R)	CTGCCTGGACAACATTGCTT			
TetB (F)	CTCAGTATTCCAAGCCTTTG	*tetB*	416	[14]
TetB (R)	CTAAGCACTTGTCTCCTGTT			
TetD (F)	ATTACACTGCTGGACGCGAT	*tetD*	1104	[14]
TetD (R)	CTGATCAGCAGACAGATTGC			

## Results

### Isolation and identification of gut bacteria

Of 122 bacterial colonies primarily selected from the culture plates, nine different bacterial genera were identified by conventional microbiological culture methods coupled with the use of analytical profile index (API) 20E^®^ system. As shown in Table 3, bacteria isolated from the gut of young children included important pathogenic bacteria along with the ones that are widely known as commensals, and were predominated by the members of the family *Enterobacteriaceae*. *Escherichia* was identified to be the dominant bacterial genera accounting for 52%, followed by *Enterobacter* (16%), *Pseudomonas* (12%), *Klebsiella* (6%), *Pantoea* (6%), *Vibrio* (3%), and *Citrobacter* (3%); while *Aeromonas* and *Raoultella* were also identified, but as infrequently occurring genera. Replicates of the bacterial colonies representing each of the genera were collected and stored in T1N1 soft agar (both at room temperature and −80 °C) for further use.

**Table 3:** Isolation of multi-drug resistant bacteria of the family Enterobacteriaceae, including the potential pathogens occurring in the gut of young children in Bangladesh

Sl.#	Bacteria	Total	Number of antibiotic resistant bacteria (%)										
			MDR	E	CN	SXT	TE	CIP	AMP	CRO	CFM	AZM	MEL
1	*E. coli*	63	52	61 (97)	9 (14)	26 (41)	45 (71)	16 (25)	42 (67)	18 (29)	28 (44)	34 (54)	7 (11)
2	*Enterobacter* spp.	19	15	19 (100)	2 (11)	13 (68)	12 (63)	0	17 (90)	10 (53)	9 (47)	8 (42)	1 (5)
3	*Pseudomonas* spp.	15	12	15 (100)	0	0	1 (7)	2 (13)	13 (87)	4 (27)	2 (13)	12 (63)	9 (60)
4	*Klebsiella* spp.	7	5	7 (100)	0	3 (43)	3 (43)	0	6 (86)	0	1 (14)	3 (43)	2 (29)
5	*Pantoea* spp.	7	4	7 (100)	1 (14)	4 (57)	5 (71)	0	5 (71)	3 (43)	3 (43)	3 (43)	1 (14)
6	*Vibrio* spp.	4	1	2 (50)	0	1 (25)	1 (25)	1 (25)	2 (40)	1 (25)	1 (25)	1 (25)	0
7	*Citrobacter* spp.	4	4	3 (75)	2 (50)	4 (100)	4 (100)	4 (100)	3 (75)	2 (50)	3 (75)	3 (75)	1 (25)
8	*Aeromonas hydrophila* gr. 1	2	1	2 (100)	0	1 (50)	1 (50)	0	0	2 (100)	1 (50)	0	0
9	*Raoultella terrigena*	1	1	1 (100)	0	0	0	0	0	0	0	1 (100)	1 (100)
10	Total	122	95 (78)	117 (96)	14 (11)	52 (43)	72 (59)	23 (19)	77 (63)	40 (33)	48 (39)	65 (53)	22 (18)

*E* erythromycin, 15 µg;* CN* gentamicin, 10 µg;* SXT* trimethoprim sulfamethoxazole, 25 µg;* TE* tetracycline, 30 µg;* CIP* ciprofloxacin, 5 µg;* AMP* ampicillin, 10 µg;* CRO* ceftriaxone, 30 µg;* CFM* cefixime, 5 µg;* AZM* azithromycin, 15;* MEL* mecillinam, 10 µg

### Antimicrobial susceptibility assay of gut bacteria

Results of antimicrobial susceptibility assay by disc diffusion method revealed 78% of the gut bacteria to be resistant to multiple antimicrobial drugs (Table 3), and were thus multidrug resistant (MDR). Among them *E. coli* was the predominant MDR (55%) bacterium followed by *Enterobacter* spp. (16%), *Pseudomonas* spp. (13%), *Klebsiella* spp. (5%), *Pantoea* spp. (4%), *Vibrio* spp. (1%), *Citrobacter* spp. (4%), *Aeromonas hydrophila* (1%) and *Raoultella terrigena* (1%).

Ca. 96% of the commensal bacteria occurring in the gut of children were resistant to erythromycin, followed by ampicillin (63%), tetracycline (59%), azithromycin (53%), sulfamethoxazole-trimethoprim (43%), cefixime (39%), and ceftriaxone (33%) (Table 3).

Since *E. coli* was the predominant MDR bacterial lineage (54%) found in the gut of young children, this bacterium was investigated for detailed drug resistance profile. Overall results revealed 3% of the MDR *E. coli* to be resistant to 9 of the 10 antimicrobial agents tested. *E. coli* strains were highly resistant to commonly used antibiotics e.g., erythromycin (97%), followed by tetracycline (71%), ampicillin (67%), azithromycin (54%), and cefixime (44%) (Table 3).

As shown in Table 3, *Enterobacter* spp. constituted the second most largest group of bacteria in the gut of young children comprising *E. cloacae*, *E. cancerogenus*, and *E. sakazakii*. Results revealed 90, 53, and 42% of the *Enterobacter* isolates to be resistant to ampicillin, ceftriaxone, and cefixime, respectively.

### Polymerase chain reaction (PCR) for detection of virulence and related marker genes

Polymerase chain reaction assay results revealed 56% of the overall gut bacteria to harbor gene cassettes namely Class 1 integron. PCR assays also revealed 11 and 22% of the *E. coli* isolates to harbor important virulence and related marker genes such as *stx*-2 and *eae,* respectively. None of the isolates was positive for *stx1*, and *lt*. *E. coli* isolates showing resistance to tetracycline carried the resistance related genes namely *tet*A (8%; 5/63), *tet*B (17.5%; 11/63), and *tet*D 6.0% (4/63). None of the other bacteria isolated from the gut of young children had any of these genes.

Resistance markers against macrolide antibiotics (erythromycin and azithromycin) e.g., *mph*A, *ere*B and *erm*B were found in 28% (34/122), 3% (3/122), and 5% (6/122) different gut bacterial isolates, respectively. The gut bacterial isolates were positive for extended spectrum betalactamase-related genes such as, *bla*_TEM_ (26%; 32/122), *bla*_SHV_ (12%; 14/122), *bla*_CMY-9_ (17%; 21/122), *bla*_CTX-M1_(32%; 39/122), *bla*_CTX-_M2 (7%; 8/122), *bla*_CMY-2_ (4%; 5/122), and *bla*_*OXA*_ (3%; 4/122) conferring resistance to β-lactam antibiotics.

### Detection of virulence and related large plasmids

**Fig. 1:**
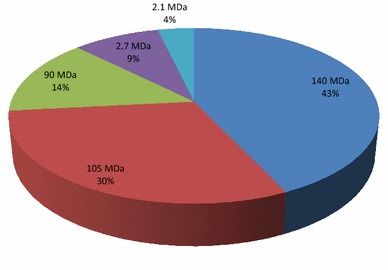
Distribution of plasmids isolated from 122 bacteria identified from fecal samples of 15 young children of moderate income families living in moderate hygiene condition in Dhaka city

Results revealed that majority of the MDR bacterial isolates from the gut of children harbored large plasmids e.g., 140 MDa (43%), 105 MDa (30%), and 90 MDa (14%) carrying invasion and related antibiotic resistance marker genes (Fig. 1).

## Discussions

Data presented in this study coupled with the data reported earlier in our culture-independent metagenomic studies provide important insights into the prevalence of MDR bacterial genera of the family Enterobacteriaceae in the gut of young children in Bangladesh. Our results appear concordant as drug resistant bacteria were reported from the gut of healthy children elsewhere [18, 19]. In Bangladesh, children suffer from frequent diarrhea and their gut can serve as an important reservoir for pathogenic bacteria of the family enterobacteriaceae, including genera that are naturally occurring in the aquatic environments [6]. The results of this culture-based gut microbiota study appear in agreement with the data obtained in our immediate past culture-independent metagenomic study showing the presence in the gut of pathogenic bacteria belonging to the family enterobacteriaceae, which included pathogenic bacteria occurring naturally in the aquatic environments [6]. Our subsequent culture-independent metagenomic studies also showed that the antimicrobial therapy in acute diarrhea results in dysbiosis, and selectively allows only the antibiotic resistant microbiota to enrich and settle in the gut of children [7]. As the course of medication ended, the prevalent antibiotic-guarded community declined, but did not disappear completely as floral restoration followed up to day 28 of our observation [7]. Thus, frequent diarrhea and related multiple doses of antibiotics likely enrich multi-drug resistant bacteria in the gut, as reflected by the observed predominance of extended-spectrum beta-lactamase (ESBL) positive Gram-negative bacterial genera including the recognized enteric and opportunistic pathogens in the gut of young children in Bangladesh. Thus, the data presented in this study might provide a basis for the widespread growth stunting related to the gut floral immaturity, as has been proposed for Bangladeshi children [20] reaching their adulthood as malnourished.

*Escherichia coli* was the most abundant bacteria as confirmed by our culture-based analysis of the gut microbiota of young children in Bangladesh. The most striking finding may be that 11% of the *E. coli* occurring in the gut of young children had genes encoding shiga toxin (*stx*)-2, and 22% possessed *eae* encoding an intimin adherence protein responsible for hemorrhagic colitis and hemolytic uremic syndrome caused by enterohemorrhagic *E. coli* (EHEC). Besides, a great majority (83%) of the *E. coli* isolated from the gut of children were MDR in Bangladesh. Drug resistant *E. coli* was reported from healthy children of USA, Venezuela, and China [21–23]; however, to our knowledge, MDR gut bacteria carrying EHEC genes namely *stx*-2 and *eae* are unique in the present study, which indicate a potential health risk for the young children of Bangladesh.

*Enterobacter cloacae*, a commensal gut bacteria occurring widely in the environment, has the ability to cause opportunistic infections in human [24, 25]. The abundance of *Enterobacter* spp., namely *E. cloacae* and *E. cancerogenus* as commensal flora of the gut of young children might reflect the potential health risks related to these opportunistic pathogens. *E. cloacae* was shown to develop resistance to cephalosporins related to high frequency mutation in the resistance genes of extended-spectrum cephalosporins and ampicillin [25–27]. In concurrence, almost half of the commensal *Enterobacter* spp. isolated from gut of young children were resistant to cephalosporin antibiotics (e.g., ceftriaxone and cefixime) in Bangladesh.

Environmental enteropathy (EE), also known as tropical enteropathy or environmental enteric dysfunction, is a condition of frequent intestinal infections [28]. There may be rare or no acute symptoms, but the chronic problem associated with the EE is the mal absorption of nutrients, which can lead to malnutrition and growth stunting in children [28]. In Dhaka city, municipal supply water was shown have multiple infectious agents, including bacteria [29]. The observed prevalence of the pathogenic member of the family Enterobacteriaceae in the gut of young children might reflect widespread environmental enteropathy in Dhaka city. Although boiling is an effective way to kill water-borne pathogens, this is often not practical due to poor or no access to fuel. A recent study revealed possible post-collection contamination of water at the household level, as the pathogen counts were much higher in the household drinking water than that of the supply water [29]. Such pathogenic bacteria causing frequent infections (3–5 episodes of diarrhea/year) can eventually settle down in the gut as a commensal microbiota [6], as we observed in the gut of children living in Dhaka city. This is plausible as the type of microbiota in the gut of cohort of European and rural African children was determined by their regular foods [30].

The observed multi-drug resistance in gut microbiota of young children of Bangladesh appeared concordant with results from studies reporting MDR bacteria from healthy fecal samples of adults [31–33] and children [18, 19, 21, 22] from other countries of the world. Plasmids are known to carry and transmit drug resistance and related genes in bacteria [33, 34]. A 140 MDa plasmid is known to be associated with the invasiveness of *Shigella flexneri* [35]. In the present study, a significant proportion (48%) of the gut bacteria of young children harbored a 140 MDa plasmids. To the best of our knowledge this is the first report of the presence of such major plasmids in bacteria other than *Shigellae* occurring in the gut of young children, which can transfer resistance and related genes horizontally across different genera.

The gene cassettes such as classes 1 and 2 integrons are responsible for multidrug resistance in Gram-negative bacteria [14]. Class 1 integron was found from 57% (39/68) of the *E. coli* occurring in the gut of young children in Dhaka city. Our results appear in agreement, as *intl*–1 was reported from as high as 71% of MDR *E. coli* isolated from human, animal, and food in Spain [14]. The observed presence of tetracycline resistance-related genes *tet*A, *tet*B, and *tet*D encoding efflux proteins in the commensal *E. coli* was in agreement in the present study, as tetracycline resistant strains have been reported from the gut of infants in a previously study conducted elsewhere [36]. Tetracycline resistance-related genes *tet*A, *tet*B, and *tet*D were found only from the MDR *E. coli* strains, but not from any other commensal bacteria occurring in the gut of young children in Bangladesh. Our results appear in agreement as *tet*A and *tet*B were present in MDR *E. coli* isolated from human, animal, and food in Spain, but all carried a wide variety of other antibiotic resistance markers [14].

Bacteria producing enzymes beta-lactamases (β-lactamases; also known as penicillinase) can degrade β-lactam antibiotics such as penicillins, cephamycins, and carbapenems (ertapenem), but rarely carbapenems, are MDR. The emergence of β-lactam resistance in *Enterobacteriaceae* is related primarily to the production of novel enzymes such as TEM-, SHV-, and OXA-type β-lactamases. Ampicillin-resistant (AMP^r^) *E. coli* isolated from foods, fecal samples of human, and healthy animals were shown to harbor bla-TEM-type β-lactamase [37]. In this study, majority (91%) of the *E. coli* isolated from the gut of young children harbored *bla*-_*TEM*_ type β-lactamases in Bangladesh. Although the remaining *E. coli* isolates possessed *bla*_OXA_ in our study, this is a clear indication of the proliferation of ESBLs producing bacteria among the commensal gut bacteria of young children, which is alarming from public health standpoint. The OXA-type β-lactamases were reported earlier in 2008 from clinical samples in Swedish hospital [38]. Previously, *E. coli* isolated from fecal samples of domestic animals, retail ground meats, and urinary samples were shown to carry and spread *bla*_CMY-2_ in southern Taiwan [39]. In the present study, two each of *Pantoea* spp., *E. cloacae*, and one *E. cancerogenus* possessed *bla*_CMY-2_. *Bla*_CMY-9_ was identified as a novel plasmid mediated cephalosporinase in *E. coli* isolated from clinical samples in Japan [40]. Our results reveal 16% (20/122) of the bacteria occurring in the gut of young children possessed *bla*_CMY-9_ gene. In addition, six *Klebsiella* spp. isolated in the present study from the gut of young children harbored the *bla*_SHV_ gene responsible for ESBL activity in *Klebsiella* spp. [41].

In our previous study in Bangladesh, we have documented that acute diarrhea expels the gut flora while antibiotic provision for the hospitalized diarrhea patients selectively allows only the MDR bacteria belonging to enterobacteriaceae to be restored in the gut of children [6]. The observed prevalence of pathogenic bacteria carrying a wide variety of drug resistance marker genes, especially the extended spectrum beta-lactamase (ESBL) suggests the gut of young (under five years) children to be an important reservoir for MDR-related genes. This is suggestive of an apparent havoc, which will make the clinical management of diarrhea and other opportunistic bacterial infections, especially in severely malnourished and immunocompromised children very challenging. It is therefore evident prudent that more children will suffer from diarrhea due to MDR bacteria in Bangladesh, a densely populated country where hygiene level is poor and the dearth of safe drinking water will invite more untreatable diseases than ever before.
